# Transmembrane emp24 domain proteins in development and disease.

**DOI:** 10.1017/S0016672319000090

**Published:** 2019-12-27

**Authors:** Rachel Aber, Wesley Chan, Sevane Mugisha, Loydie A. Jerome-Majewska

**Affiliations:** 1 Department of Anatomy and Cell Biology, McGill University, Montreal, Quebec, Canada; 2 Department of Pharmacology and Therapeutics, McGill University, Montreal, Quebec, Canada; 3 Department of Human Genetics, McGill University, Montreal, Quebec, Canada; 4 Department of Pediatrics, McGill University, Montreal, Quebec, Canada

**Keywords:** cargo receptor, development, disease, p24, TMED

## Abstract

Regulated transport through the secretory pathway is essential for embryonic development and homeostasis. Disruptions in this process impact cell fate, differentiation and survival, often resulting in abnormalities in morphogenesis and in disease. Several congenital malformations are caused by mutations in genes coding for proteins that regulate cargo protein transport in the secretory pathway. The severity of mutant phenotypes and the unclear aetiology of transport protein-associated pathologies have motivated research on the regulation and mechanisms through which these proteins contribute to morphogenesis. This review focuses on the role of the p24/transmembrane emp24 domain (TMED) family of cargo receptors in development and disease.

## 1. Introduction

**Fig. 1.**
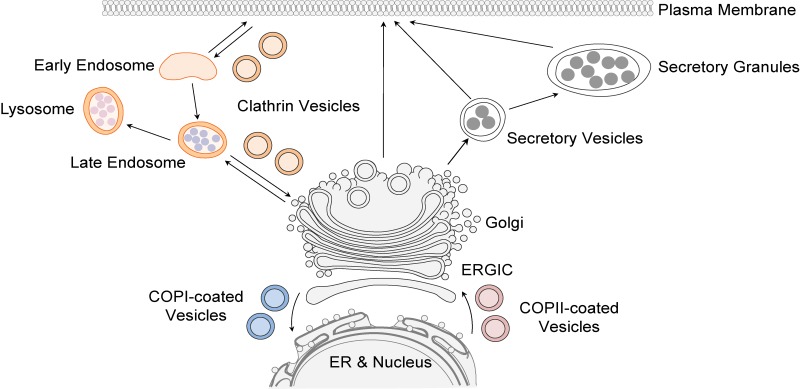
Summary of the secretory pathway. Transmembrane and secreted proteins are folded in the endoplasmic reticulum (ER) and transported to the Golgi via COPII-coated vesicles (anterograde trafficking). ER-resident or misfolded proteins are trafficked back to the ER from the Golgi via COPI-coated vesicles (retrograde trafficking). Clathrin-coated vesicles mediate a portion of post-Golgi trafficking. ERGIC = ER–Golgi intermediate compartment.

**Fig. 2.**
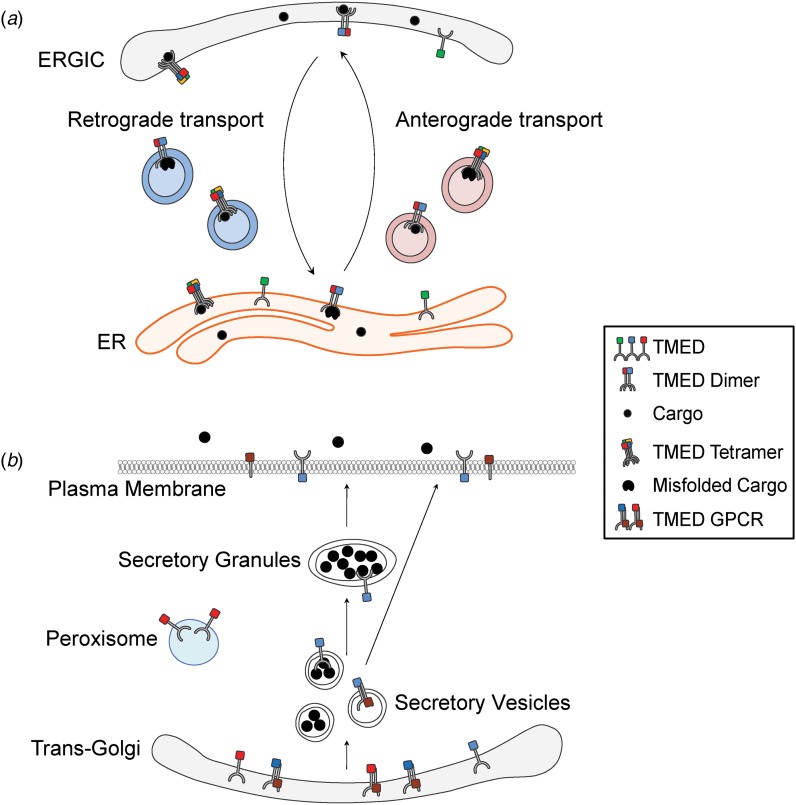
TMED proteins in the secretory pathway. (a) TMED dimers and tetramers are packaged into COPII-coated vesicles (pink) and COPI-coated vesicles (blue) and are implicated in anterograde and retrograde transport. (b) A subset of TMED proteins are also found at the plasma membrane, in secretory granules, at the *trans*-Golgi and in peroxisomes. ER = endoplasmic reticulum; ERGIC = ER–Golgi intermediate compartment; GPCR = G-protein-coupled receptor.

Nascent proteins are modified and transported to their final destination via the secretory pathway (Figure 1). Both regulated and constitutive transport of these cargo proteins are essential for embryonic development. In fact, a number of human diseases, including congenital malformations and cancers, arise as a result of mutated or abnormal expression of proteins in this pathway (Merte *et al.*, 2010; Routledge *et al.*, 2010; Garbes *et al.*, 2015; Yehia *et al.*, 2015; Zhang *et al.*, 2017). The transmembrane emp24 domain (TMED) family consists of type I single-pass transmembrane proteins that are found in all eukaryotes. Members of the TMED family have emerged as important regulators of protein transport (Carney & Bowen, 2004; Dancourt & Barlowe, 2010; Jerome-Majewska *et al.*, 2010; Zakariyah *et al.*, 2012; Viotti, 2016; Wada *et al.*, 2016; Hou *et al.*, 2017; Hou & Jerome-Majewska, 2018), and although they are found in the early and late secretory pathways (Figure 2), most studies focus on the function of TMED proteins in the early secretory pathway (Figure 2a): the endoplasmic reticulum (ER), the ER–Golgi intermediate compartment (ERGIC) and the Golgi (Shevchenko *et al.*, 1997; Dominguez *et al.*, 1998; Fullekrug *et al.*, 1999; Jenne *et al.*, 2002; Chen *et al.*, 2006; Hosaka *et al.*, 2007; Blum & Lepier, 2008; Montesinos *et al.*, 2012, 2013).

**Fig. 3.**
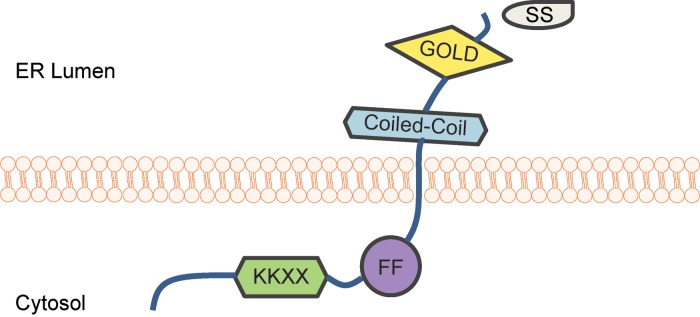
Domain structure of TMED family proteins. TMED proteins have a signal sequence (SS) that enables their translocation into the endoplasmic reticulum (ER); the SS is cleaved following ER translocation. The luminal portion of TMED proteins consists of a coiled-coil domain and a Golgi dynamics (GOLD) domain. The short cytoplasmic tail includes diphenylalanine (FF) and dilysine motifs (KK), which are important for binding to COP proteins.

Members of the TMED family are classified into four subfamilies based on sequence homology (Table 1): *α*, *β*, *γ* and *δ* (Blum *et al.*, 1996; Strating *et al.*, 2009; Schuiki & Volchuk, 2012; Pastor-Cantizano *et al.*, 2016). However, as the different TMED subfamilies have expanded independently during evolution, the number of proteins in each subfamily varies by species (Table 1) (Strating *et al.*, 2009). Nonetheless, all TMED family members share a common structural organization (Figure 3): a luminal Golgi-dynamics (GOLD) domain, a luminal coiled-coil domain, a transmembrane domain and a short cytosolic tail (Strating *et al.*, 2009; Nagae *et al.*, 2016, 2017; Pastor-Cantizano *et al.*, 2016).

**Table 1.** TMED family orthologues across different organisms.

Subfamily	Human/mouse (HGCN)	*Drosophila (FlyBase)*	Yeast	*Caenorhabditis elegans (WormBase)*	*Xenopus (Xenbase)*
*α*	*TMED4*	*éclair*	Erp6	*tmed-4*	*tmed4*
		*p24-2*			
	*TMED9*		Erp5		*tmed9*
				*tmed-12*	
	*TMED11*		Erp1		
*β*	*TMED2*	*CHOp24*	Emp24	*sel-9*	*tmed2*
		*CG9308*			
*γ*	*TMED1*			*tmed-1*	*tmed1*
	*TMED3*	*p24-1*	Erp4	*tmed-3*	
	*TMED5*	*opossum*	Erp2		*tmed5*
				*tmed-13*	
		*CG31787*			
	*TMED6*	*logjam*			*tmed6*
	*TMED7*		Erp3		*tmed7*
*δ*	*TMED10*	*baiser*	Erv25	*tmed-10*	*tmed10*
					*tmed10-like*

During anterograde and retrograde transport, TMED proteins dimerize and interact with coatomer (COP) protein complexes to facilitate cargo selection and vesicle formation. The GOLD domain, which was expected to be involved in cargo recognition (Anantharaman & Aravind, 2002), was recently shown to mediate dimerization between TMED proteins (Nagae *et al.*, 2016, 2017); a function previously ascribed to the coiled-coil domain. The transmembrane domain, which was predicted to mediate interactions with integral membrane proteins in order to regulate ER exit (Fiedler & Rothman, 1997), was more recently shown to interact with lipids and to aid in vesicle budding (Contreras *et al.*, 2012; Ernst & Brügger, 2014; Aisenbrey *et al.*, 2019). Interactions with COPI and COPII occur via conserved COP binding motifs in the cytoplasmic tail (Figure 3) (Dominguez *et al.*, 1998; Majoul *et al.*, 2001).

TMED proteins regulate transport of a diverse group of cargo proteins; these are summarized in Tables 2 and 3, respectively, depending on whether direct interaction with TMED proteins has or has not been shown (Anantharaman & Aravind, 2002; Theiler *et al.*, 2014; Nagae *et al.*, 2016, 2017). The best-characterized TMED cargo proteins are glycosylphosphatidylinositol (GPI)-anchored proteins (Marzioch *et al.*, 1999; Muniz *et al.*, 2000; Takida *et al.*, 2008; Bonnon *et al.*, 2010; Castillon *et al.*, 2011; Fujita *et al.*, 2011; Theiler *et al.*, 2014; Pastor-Cantizano *et al.*, 2016). However, members of the TMED family also interact with transmembrane and secreted proteins such as Notch and G-protein-coupled receptors, as well as with a number of Wnt ligands (Tables 2 & 3) (Stirling *et al.*, 1992; Marzioch *et al.*, 1999; Wen & Greenwald, 1999; Luo *et al.*, 2007, 2011; Stepanchick & Breitwieser, 2010; Buechling *et al.*, 2011; Port *et al.*, 2011; Junfeng *et al.*, 2015; Li *et al.*, 2015). In this review, we summarize the growing body of literature indicating requirements for TMED proteins during embryogenesis and their contributions to disease.

**Table 2.** Cargo interactors of TMED proteins.

TMED protein	Cargo	Interaction location	Experimental system
TMED1	IL1RL1 (ST2L/IL-33R)	ER, *cis*-Golgi	Cell culture
TMED2	AGR2	n/a	Cell culture and mouse
	GCGR	n/a	Cell culture
	TMEM173 (MITA)	ER	Cell culture
	Wg^*a*^	ER and Golgi	*Drosophila*
	CD59^*a*^	ER	Cell culture
	Folate receptor^*a*^	ER	Cell culture
	Gas1p^*a*^	ER	Yeast
	F2R (PAR-1)	Golgi	Cell culture
	F2RL1 (PAR-2)^*a*^	Golgi	Cell culture
	P2YR (1,2,4,11)	Golgi	Cell culture
	OPRM1 (MOR1B)	Golgi	Cell culture
	CASR	ER	Cell culture
	SM-18	COPI vesicles	Cell culture
TMED9	PS1-NTF^*a*^	n/a	Cell culture
	NCT^*a*^	n/a	Cell culture
	APH-1^*a*^	n/a	Cell culture
	PEN-2^*a*^	n/a	Cell culture
TMED10	TGFBR2	Cell membrane	Cell culture
	TGFBR1 (ALK5)	Cell membrane	Cell culture

^*a*^ Also interacts with TMED10.

ER = endoplasmic reticulum; n/a = not applicable.

**Table 3.** Proteins regulated by the TMED family.

TMED protein	Target protein	Regulation type	Experimental system
TMED1	Wg^*a*,*b*^	Secretion	*Drosophila*
TMED2	Invertase^*c*^	Secretion	Yeast
	Kar2p^*c,d*^	Secretion	Yeast
	Sec13^*c,d*^	–	Yeast (genetic interaction)
	LIN-12/GLP-1^*c*^	Localization	*Caenorhabditis elegans* (genetic interaction)
	Fibronectin	Localization	Mouse
	VCAM1	Localization	Mouse
TMED3	WNT1	Localization, secretion	Cell culture
TMED4	POMC^*c*^	Processing	*Xenopus*
TMED5	Gas1p^d^	Secretion, processing	Yeast
	WntD	Secretion	*Drosophila*
TMED6	Insulin^*c*^	Secretion	Cell culture
TMED7	TLR-4	Signalling	Cell culture
TMED9	APP	Processing	Cell culture
TMED10	CD55 (DAF)	Processing	Cell culture
	P2YR (4)	Localization	Cell culture
	OPRM1 (MOR1B)	Localization	Cell culture
	TMEM173 (MITA)	Signalling	Cell culture
	Tkv^*a*^	–	*Drosophila* (genetic interaction)

^*a*^ Also regulated by TMED4.

^*b*^ Also regulated by TMED5.

^*c*^ Also regulated by TMED10.

^*d*^ Also regulated by TMED11.

## 2. TMED proteins and development

**Fig. 4.**
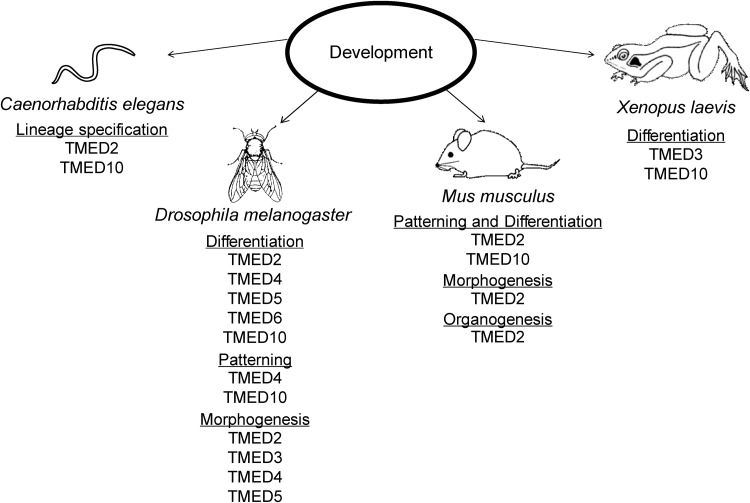
TMED family in development. TMED proteins regulate multiple developmental processes in different organisms.

Although the TMED family is essential for normal development, with limited functional redundancy (Strating *et al.*, 2007, 2009), the specific functions of most TMED proteins are unclear. In this section, we highlight the roles uncovered for TMED proteins during development in a variety of species (summarized in Figure 4). Mutant *Tmed* alleles are summarized in Table 4.

**Table 4.** Mutant Tmed alleles in various model organisms.

Tmed member	Mutation type	Allele/mechanism	System	Reference
*Sel-9* (tmed2)	Point mutations	Multiple alleles: p.V47L, p.G51R, p.G51E, p.S97N, p.W174X/antimorphic and hypomorphic	*Caenorhabditis elegans*	Wen and Greenwald (1999)
*Tmed2*	Point mutation	p.A13E/protein null	Mouse	Jerome-Majewska *et al.* (2010)
	*β-galactosidase* insertion (gene trap)	*Tmed2*-*β*-geo fusion/antimorphic	Mouse	
*Loj* (tmed6)	*P*-element insertion in 5′-untranslated region and exon 2	RNA null	*Drosophila*	Carney and Taylor (2003)
*Eca* (tmed4)	Premature stop codon	pW201X/antimorphic, null	*Drosophila*	Bartoszewski *et al.* (2004)
	Deletion (300 nt)	pS7delfsx/null	*Drosophila*	
*Bai* (tmed10)	Point mutation at splice site	pG124fsx/null	*Drosophila*	
*Tmed10*	Exon 1 deletion	RNA/protein null	Mouse	Denzel *et al.* (2000)

### 2.1. Caenorhabditis elegans

The *Caenorhabditis elegans* genome contains at least one representative member of all four TMED subfamily members (WormBase; WS270) (Table 1): there are two genes in the *α* subfamily (*tmed-4* (Y60A3.9) and *tmed-12* (T08D2.1)), one gene each in the *β* subfamily (*sel-9* (*tmed-2*; W02D7.7)) and in the *δ* subfamily (*tmed-10* (F47G9.1)) and three genes in the *γ* subfamily (*tmed-1* (K08E4.6), *tmed3* (F57B10.5) and *tmed-13* (Y73B6BL.36)). Two *tmed2* mutant alleles were first isolated as suppressors of the egg-laying defect associated with hypomorphic alleles of *lin-12* (Table 4) (Sundaram & Greenwald, 1993). Wen and Greenwald isolated five additional *tmed2* mutant alleles in a non-complementation screen (Table 4) (Wen & Greenwald, 1999) and suggested that all seven *sel-9* alleles were antimorphic (dominant negative) and reduced wild-type *sel-9* activity. Interestingly, six of these mutations mapped to the GOLD domain and one mutation was predicted to generate a truncated protein lacking the carboxyl region. Furthermore, though RNA interference (RNAi) knockdown of *sel-9* and *tmed10* did not affect viability, a truncated mutant allele of *sel-9* resulted in phenotypes classified as dumpy (Dpy), uncoordinated (Unc), roller (Rol) and defective egg-laying (Wen & Greenwald, 1999). Reduced wild-type SEL-9 and TMED-10 activity allowed mutated GLP-1 protein to reach the plasma membrane and resulted in increased activity of mutated LIN-12 or GLP-1. Thus, although null alleles for *tmed* genes have not yet been examined in *C. elegans*, the phenotypes associated with mutations in *sel-9* suggest a role for this family in quality control during LIN-12/Notch receptor family protein transport. These studies further indicate that the GOLD domain may play an important role in mediating interactions with LIN-12 and GLP-1.

### 2.2. Drosophila melanogaster

Nine TMED genes have been identified in the *Drosophila* genome (FlyBase; FB2019_03) (Table 1): two *α* (p24-2 and éclair), two *β* (CG9308 and CHOp24), one *δ* (baiser) and four *γ* (p24-1, logjam, opossum and CG31787)). Most of these TMED proteins are localized to the ER–Golgi interface (Boltz *et al.*, 2007; Buechling *et al.*, 2011; Saleem *et al.*, 2012). *Logjam* (*Tmed6*, *loj*), *CHOp24* (*Tmed2*), *p24-1*(*Tmed3*), *opossum* (*Tmed*5, *opm*), *eclair* (*Tmed4*, *eca*) and *baiser* (*Tmed10*, *bai*) were expressed at all developmental stages and in adult tissues (Carney & Taylor, 2003; Boltz *et al.*, 2007; Graveley *et al.*, 2011). *CG31787* and *CG9308* were expressed in a sex-dependent pattern and *p24-2* was primarily expressed in adult tissues with limited or no expression in pupal and larval tissues, respectively (Boltz *et al.*, 2007). Expression of a subset of *Tmed* genes during development (*Tmed2*, *Tmed3*, *Tmed4*, *Tmed5*, *Tmed6* and *Tmed10*) suggests that they may be involved in *Drosophila* development beginning at the earliest stages of embryogenesis.

Mutations in *Drosophila* TMED family members (Table 4) and RNAi experiments indicate a role for TMED proteins in reproduction, embryonic patterning, transforming growth factor-*β* (TGF-*β*) signalling and WNT signalling (Carney & Taylor, 2003; Bartoszewski *et al.*, 2004; Buechling *et al.*, 2011; Port *et al.*, 2011; Li *et al.*, 2015). P-element insertions in the 5′-untranslated region or exon 2 of *loj*, the first coding exon, were shown to result in a loss of *loj* (*Tmed6*) RNA. These mutations led to oviposition defects in female *Drosophila* (Table 4) (Carney & Taylor, 2003) and revealed a requirement for this protein in the central nervous system and the mature egg. Bartoszewski and colleagues reported mutations in two additional *Drosophila* family members (Bartoszewski *et al.*, 2004). Deletion of a large portion of the coding region of *eca* (*tmed4*) or a point mutation leading to a premature stop codon before the carboxyl domain of the protein were phenotypically indistinguishable, indicating that the carboxyl domain is essential for *eca* function. The group also identified mutants with a splice-site mutation in *bai* (tmed10), which led to a premature stop codon and a null allele. They showed that *eca* was required for both TGF signalling and for Wg secretion. *eca* and *bai* mutants had reduced survival, and surviving mutants showed fertility defects; similar to *loj* mutants, males had reduced fertility and females did not lay eggs. In addition, *eca* and *bai* genetically interacted and were both required in oocytes for dorsal–ventral patterning of embryos (Table 4) (Bartoszewski *et al.*, 2004). This phenotype was specific for maternally deposited TKV, an orthologue of the mammalian TGF-*β* receptor BMPR1, and downstream of Dorsal, indicating that the two TMED proteins are required for activity of maternally deposited *Drosophila* BMPR1. Furthermore, although *bai* mutants phenocopied the temperature-sensitive thick vein phenotype of *tkv* mutants, it was not clear why reduced levels of TMED proteins blocked signalling by this TGF-*β* receptor, especially since the TKV protein was still properly localized in these mutants. Altogether, studies in *Drosophila* indicate that normal levels of tmed proteins are required for morphogenesis and patterning.

Multiple studies support a role for TMED proteins in WNT signalling. In an RNAi screen, *eca* and *CHOp24* were found to be required for Wg transport (Port *et al.*, 2011). Knockdown of these two genes resulted in loss of wing margin tissue, retention of Wg in the ER and reduced extracellular Wg. In a similar screen, Buechling *et al.* found that *opm*, *CHOp24* and *p24-1* were required in Wg-producing cells for canonical Wg signalling (Buechling *et al.*, 2011). Furthermore, the group showed that maternal deposits of Opm were required for viability and WntD transport. In a more recent RNAi screen, Li *et al.* identified *bai*, *CHOp24* and *eca* to be important in Wg-producing cells (Li *et al.*, 2015). All three studies suggest that TMED family members are implicated in the transport of WNT proteins from the ER to the Golgi. Furthermore, *eca* and *bai* are required for both TGF and WNT signalling at different developmental stages and in different tissues, suggesting that TMED proteins function in multiple pathways and are regulated both temporally and spatially.

### 2.3. Xenopus laevis

The *Xenopus tmed* gene family is composed of nine members (Xenbase) (Table 1): two *δ* subfamily members (*tmed10* and *tmed10-like*) (Strating *et al.*, 2009), four *γ* subfamily members (*tmed1*, *tmed5*, *tmed6* and *tmed7*), two *α* subfamily members (*tmed4* and *tmed9*) and one *β* subfamily member (*tmed2*). The TMED family is involved in *X. laevis* pro-opiomelanocortin (POMC) protein biosynthesis in melanotrope cells of the intermediate pituitary gland. POMC is a precursor polypeptide that is cleaved to produce multiple peptide hormones. Six of the Tmed proteins (Tmed2, Tmed4, Tmed5, Tmed7, Tmed10 and Tmed10-like) are expressed in melanotrope cells, whereas the other two members (Tmed1 and Tmed9) are not (Rotter *et al.*, 2002). Tmed proteins are generally localized to the Golgi compartment in *Xenopus*. However, the steady-state subcellular localization shifts towards a subdomain of the ER and ERGIC in biosynthetically active intermediate pituitary melanotrope cells (Kuiper *et al.*, 2001). Furthermore, Tmed2, Tmed4, Tmed7 and Tmed10-like are upregulated in active melanotropes compared to inactive melanotropes (Rotter *et al.*, 2002).

Tmed proteins co-localize with the major cargo protein POMC and have functionally non-redundant roles in regulating its secretion. For example, transgenic expression of Tmed4 dramatically reduces POMC transport, leading to its accumulation in ER-localized electron-dense structures (Strating *et al.*, 2007). By contrast, transgenic expression of Tmed10-like does not induce changes in the cell's internal structure or in POMC secretion, but it does impact Golgi-based processing of POMC (Strating *et al.*, 2007). Furthermore, transgenic expression of Tmed4 or Tmed10 disrupted pigmentation in *Xenopus* because of insufficient POMC secretion or abnormal POMC processing, respectively (Bouw *et al.*, 2004; Strating *et al.*, 2007). The *Xenopus* POMC studies are critical indications that Tmed4 and Tmed10 can affect different sub-compartments in the early stages of the secretory pathway and can be localized differentially depending on cell type (Kuiper *et al.*, 2000). These studies suggest an emerging model where Tmed2, Tmed4, Tmed7 and Tmed10 function together (potentially as a tetramer) to traffic POMC through the early secretory pathway during pigment development. Since POMC neurons are also found in mammals, TMED proteins may play an evolutionarily conserved role in the development of the melanocortin system in humans.

### 2.4. Mouse/human

There are ten known *TMED* genes in mammals (Table 1): three *α* subfamily (*Tmed4*, *Tmed9* and *Tmed11*), five *γ* subfamily (*Tmed1*, *Tmed3*, *Tmed5*, *Tmed6* and *Tmed7*), one *β* subfamily (*Tmed2*) and one *δ* subfamily (*Tmed10*), with an additional non-functional duplicate of *Tmed10* found solely in apes and humans (Blum *et al.*, 1996; Horer *et al.*, 1999). Individual TMED proteins appear to be more crucial in mammals during embryonic development when compared to less complex organisms. We generated two mutant mouse lines with mutations in *Tmed2*; one line carries a single point mutation in the signal sequence of *Tmed2* and the second line has a *β-galactosidase* gene trap inserted in intron 3 of the gene. Both mutations resulted in a loss of TMED2 protein and embryonic lethality by embryonic day (E) 11.5 (mid-gestation) (Table 4) (Jerome-Majewska *et al.*, 2010). Similarly, deletion of exon 1 of mouse *Tmed10* results in loss of TMED10 protein and embryonic lethality at E3.5, before implantation (Denzel *et al.*, 2000). Thus, TMED2 and TMED10 have non-redundant functions during embryogenesis.

*Tmed2* mRNA is expressed in a temporal and a tissue-specific pattern in mice (Jerome-Majewska *et al.*, 2010). At E5.5, *Tmed2* is expressed in embryonic and extraembryonic regions. Expression becomes higher in the ectoplacental cone and extraembryonic ectoderm at E6.5. At E7.5, *Tmed2* is observed in the ectoplacental cone, amnion, anterior neural folds and head mesoderm. At E8.5, *Tmed2* expression is found in the somites, heart and tail bud. Prior to lethality by E11.5, *Tmed2* homozygous null mutants are developmentally delayed (beginning at E8.5) and present with an abnormally looped heart (E9.5) and an abnormal tail bud morphology (E9.5–E10.5) (Jerome-Majewska *et al.*, 2010). Therefore, TMED2 is important for morphogenesis of the organs in which it is expressed.

In addition, *Tmed2* is essential for the formation of the placenta (Jerome-Majewska *et al.*, 2010). At E8.5, *Tmed2* mRNA is expressed in the allantois, chorionic plate and giant cells. At E9.5, *Tmed2* continues to be expressed in the giant cells, spongiotrophoblasts and the forming labyrinth layer, but it is no longer expressed in the allantois. At E10.5, *Tmed2* expression is similar to E9.5; however, expression in giant cells is restricted to a subset of the layer and expression can be observed in the maternal decidua. In accordance with the expression of *Tmed2* in the placenta, E9.5–E10.5 placentas of *Tmed2* homozygous mutants fail to form a labyrinth layer, resulting in lethality at mid-gestation, with a subset of embryos failing to undergo chorioallantoic attachment (Jerome-Majewska *et al.*, 2010). TMED2's requirement in the placenta is cell-autonomous in the chorion and non-cell-autonomous in the allantois where it regulates fibronectin and VCAM1 localization during chorioallantoic attachment (Hou & Jerome-Majewska, 2018). These studies indicate a crucial role for TMED2 in the development of the placenta. Furthermore, TMED2 is expressed in the human placental syncytiotrophoblast, cytotrophoblast and stromal cells between the gestational ages of 5.5 and 40 weeks (Zakariyah *et al.*, 2012). Thus, TMED2 may also be crucial during human placental development.

TMED10 is expressed in the embryonic mouse brain at E15 and expression declines with age in the developing postnatal brain (Vetrivel *et al.*, 2008). In the rat central nervous system, TMED10 expression is found in the cortex, hippocampus and brainstem at postnatal day (P) 7 and P21 (Vetrivel *et al.*, 2008). TMED10 is also expressed in the developing postnatal sensory epithelium of the murine inner ear at P3, but not at P14 and P30, suggesting that TMED10 may play a role in inner ear development (Darville & Sokolowski, 2018). The role, if any, of TMED10 in the pre- and post-natal development of the mammalian brain and inner ear remains to be identified.

Overall, TMED proteins are expressed in a tissue-dependent, developmental age-dependent and even sex-specific pattern, suggesting non-redundant functions across various species (Rotter *et al.*, 2002; Carney & Taylor, 2003; Boltz *et al.*, 2007; Jerome-Majewska *et al.*, 2010; Hou *et al.*, 2017). The TMED family is implicated in developmental processes such as wing morphogenesis in *Drosophila*, reproductive system development in *C. elegans*, embryonic development in rats and mice and placental development in mice and humans (Jerome-Majewska *et al.*, 2010; Buechling *et al.*, 2011; Hou & Jerome-Majewska, 2018). Furthermore, the role for TMED5 and TMED10 in regulating WNT and TGF-*β* signalling, respectively, seems to be evolutionarily conserved in *Drosophila* and humans (Buechling *et al.*, 2011; Port *et al.*, 2011). In addition, components of the Notch family of receptors could also be an evolutionarily conserved cargo of the TMED family. Our growing understanding of the involvement of the TMED family in mammalian development paves the foundation for understanding their potential roles in human diseases.

## 3. TMED proteins and disease

**Fig. 5.**
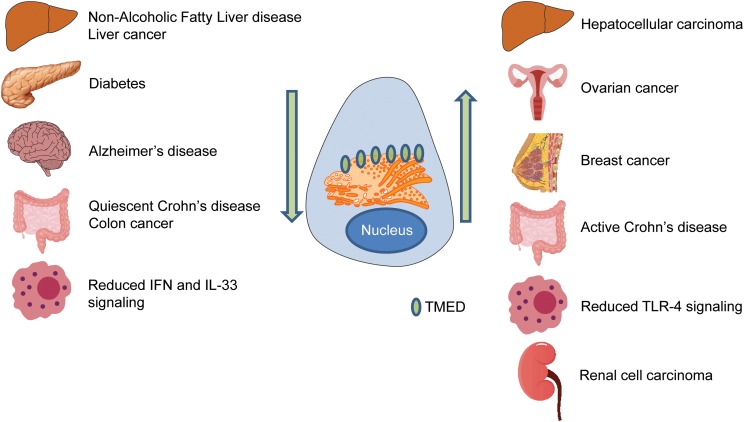
TMED family in disease. Disrupted TMED protein levels are associated with a diverse range of diseases. Arrows indicate levels of TMED protein within the cell. Organs represent different diseases associated with TMED proteins. IFN = interferon; TLR = Toll-like receptor.

Abnormalities in the controlled transport of proteins in the secretory pathway contribute to diseases such as cancer. More specifically, aberrant expression of TMED proteins is implicated in cancer, liver disease, pancreatic disease and immune system dysregulation. Although these diseases differ greatly in symptom presentation, they reveal the importance of balanced levels of TMED proteins within cells (Figure 5). Excess TMED within the cell can cause a similar phenotype as having too little TMED protein, which makes this family challenging to study.

### 3.1. TMED proteins and cancer

Current research on TMED proteins suggests an important role in cell proliferation and differentiation, especially with regards to malignancy. TMED2, the sole member of the mammalian *β* family, displays a cell type-specific role in cancer (Xiong *et al.*, 2010; Shi-peng *et al.*, 2017). In liver cells, TMED2 is required for normal cell proliferation. The aforementioned homozygous null mouse embryos, with no functional TMED2, were smaller than their littermates prior to death at mid-gestation, suggesting a requirement for TMED2 in normal cell proliferation (Table 4) (Jerome-Majewska *et al.*, 2010). Moreover, heterozygotes were more susceptible to liver cancer compared to their wild-type littermates (Hou *et al.*, 2017). Therefore, in liver cells, a reduction in TMED2 results in increased liver tumorigenesis and demonstrates a possible tumour suppressor-like property for TMED2. In contrast, elevated levels of TMED2 were found in ovarian carcinoma patients (Shi-peng *et al.*, 2017), and ectopic expression of TMED2 in ovarian cancer cells resulted in increased cell proliferation and cell migration, characteristic of metastatic cells. Furthermore, Xiong and colleagues reported that ectopic expression of TMED2 accelerates cell proliferation in murine bone cells, MC3T3-E13 (Xiong *et al.*, 2010). Elevated expression of TMED2 in breast cancer patients was identified as an unfavourable prognostic factor (Lin *et al.*, 2019). Thus, depending on cell type, TMED2 may exert either tumour suppressor or oncogenic properties.

Much like TMED2, the role of TMED3 in cancer is cell type-specific. TMED3 expression is upregulated in a number of tumours. TMED3 was identified as a prognostic biomarker of renal cell carcinoma, since higher TMED3 expression levels were found in high-stage and -grade cohorts when compared to low-stage and -grade cohorts (Ha *et al.*, 2019). TMED3 was also identified as a potential drug target for prostate cancer since it is elevated in patient tumour samples (Vainio *et al.*, 2012). In addition, TMED3 expression is upregulated in human hepatocellular carcinoma tissues. Its expression was highest in metastatic hepatocellular carcinomas when compared to non-metastatic tumours. In cell culture, TMED3 promotes the proliferation, migration and invasion of breast cancer cells, MCF-7 and MDA-MB-231 (Pei *et al.*, 2019). In fact, TMED3 knockdown inhibited hepatocellular carcinoma cell migration, whereas TMED3 overexpression enhanced cell motility. In liver cells, it is thought to promote metastasis through the IL-11/STAT3 signalling pathway, as both IL-11 and STAT3 phosphorylation levels are elevated in cells overexpressing TMED3 (Zheng *et al.*, 2016).

However, although TMED3 seems oncogenic in hepatocellular carcinomas, renal cell carcinomas, prostate cancers and breast cancers, it appears to have tumour suppressor properties in human colon cancers. In fact, TMED3 was recovered from a genome-wide *in vivo* screen for metastatic suppressors in human colon cancer (Duquet *et al.*, 2014). When TMED3 was knocked down in colon cancer cells, TMED9 was upregulated more than twofold. TMED3-mediated WNT signalling is proposed to inhibit metastasis by repressing TMED9. In the same study, loss of TMED3 resulted in increased TMED9 levels and was correlated with increased TGF-*β* signalling and upregulation of genes with migratory and invasive roles, while also repressing WNT signalling (Mishra *et al.*, 2019). Thus, similarly to TMED2, the malignant properties of TMED3 are cell type-specific. Although TMED9 has not been investigated in other cancers, in colon cancer it functions as an oncogene.

TMED10 has an important role in limiting TGF-*β* signalling. TGF-*β* is a secreted cytokine that modulates cell proliferation, differentiation and apoptosis, and it is implicated in numerous cancers. TMED10 regulates the dissociation of the TGF-*β* heteromeric I/II receptor complex. In fact, an isolated small peptide derived from the extracellular domain of TMED10 is sufficient to antagonize TGF-*β* signalling, which could be used as a therapy for cancers with abnormal TGF-*β* signalling activity (Nakano *et al.*, 2017).

Our understanding of the contribution of disrupted TMED levels in cancer is in its infancy. However, it appears that their role is tissue- and context-dependent. Since, TMED proteins may promote or inhibit cancer onset and progression, it is important to identify the specific contributions of individual TMED proteins. Intriguingly, TMED proteins may also form regulatory loops to regulate each other's levels during cancer progression.

### 3.2. Dysregulated immune responses

TMED1 interaction with ST2L, a member of the IL-R family, regulates signalling of IL-33, a proinflammatory cytokine. Following TMED1 knockdown, production of IL-6 and IL-8 – downstream targets of IL-33 – was impaired, indicating that the absence of TMED1 reduced IL-33 signalling. Surprisingly, the interaction between TMED1 and ST2L was found to occur via a protrusion of the GOLD domain of TMED1 into the cytosolic compartment (Connolly *et al.*, 2013).

TMED2 is necessary for cellular interferon (IFN) responses to viral DNA. MITA (mediator of IRF3 activation also known as STING) has a vital role in innate immune responses to cytosolic viral dsDNA. The luminal domain of TMED2 associates with MITA and stabilizes dimerization of MITA following viral infection. Association with MITA facilitates translocation into the ER, as well as anterograde trafficking from the ER to the Golgi. Consequently, suppression or deletion of TMED2 in cells markedly increased titre volume of herpes simplex virus 1 (HSV-1) and led to impaired IFN-1 production upon HSV-1 infection. Moreover, although TMED10 knockdown led to reduced CXCL10 levels, it did not interact directly with MITA and did not disrupt the synergy of TMED2 on cGAS-MITA-mediated activation of an IFN-stimulated response element. Thus, TMED2 is essential for IFN responses to viral DNA, and it is required for the translocation, localization and dimerization of MITA (Sun *et al.*, 2018).

Interestingly, TMED2 was recently reported to play a role in Crohn's disease through the regulation of the dimeric state of AGR2 (anterior gradient 2) (Maurel *et al.*, 2019). Dimeric AGR2 protein acts as a sensor of ER homeostasis. In the presence of ER stress, AGR2 dimers are disrupted and AGR2 monomers are secreted to induce inflammation in the cell. Secreted AGR2 monomers may act as a systemic alarm signal for proinflammatory responses. When TMED2 levels are lower in the cell, partial homeostasis is achieved, resulting in some inflammation from the increase in monomeric AGR2. Thus, low levels of TMED2 are associated with quiescent Crohn's disease as a consequence of fewer AGR2 dimers, causing some inflammation. However, when TMED2 levels are high in the cell, homeostasis is entirely disrupted, resulting in severe acute inflammation (active Crohn's disease). In fact, Crohn's patients with severe acute inflammation had high levels of TMED2 and disrupted autophagy (Maurel *et al.*, 2019). Since the dimeric state of AGR2 is regulated by TMED2, stable levels of TMED2 in the cell may work to suppress inflammatory responses.

TMED7, a member of the *γ* family, has a critical role in the negative regulation of TLR4 (Toll-like receptor) signalling. Following lipopolysaccharide (LPS) stimulation of human monocytes, TMED7 shows a biphasic increase. Moreover, TMED7 localized mostly in the Golgi and in endosomes, with increased localization to late endosomes following LPS stimulation. Homotypic interaction between TMED7 and TAG disrupts the formation of the TRIF/TRAM complex, inhibiting the MyD88-independent TLR4 signalling pathway. Thus, TMED7 may be a critical inhibitor of TLR4 signalling and innate immune signalling (Doyle *et al.*, 2012).

It is noteworthy that only TMED7 and TMED1 have their GOLD domains in the cytosol (Doyle *et al.*, 2012; Connolly *et al.*, 2013). No other TMED protein has been shown to have a cytosolic GOLD domain, suggesting that this may be a unique feature of these two *γ* family members.

### 3.3. Pancreatic disease

An insulinoma is a rare neuroendocrine insulin-secreting tumour derived from pancreatic *β* cells. Most insulinomas are benign in that they grow exclusively at their site of origin, but a minority metastasize. The aforementioned TMED10 and pancreatic secretory granules study showed that TMED10 is localized to the plasma membrane of pancreatic *β* cells, where it is involved in the quality control, folding and secretion of insulin molecules (Blum *et al.*, 1996; Hosaka *et al.*, 2007; Zhang & Volchuk, 2010). Knockdown of TMED10 markedly impaired insulin biosynthesis and release; however, the mechanisms by which this occurs are still unclear. In fact, knockdown of TMED10 does not impact total protein levels, suggesting that the effects of TMED10 on insulin biosynthesis are independent of its other secretory pathway functions.

TMED6 has also been implicated in diabetes. Its expression was significantly lower in diabetic rats (Wang *et al.*, 2012), and knockdown of TMED6 in mouse pancreatic Min6 *β* cells resulted in decreased insulin secretion (Fadista *et al.*, 2014). TMED6 expression in pancreatic islets of patients with type 2 diabetes was reduced when compared to control islets isolated from healthy patients. Thus, TMED6 seems also to be required for normal pancreatic function and insulin secretion (Fadista *et al.*, 2014).

### 3.4. Dementia and Alzheimer's disease

Alzheimer's disease is characterized by the extracellular accumulation of amyloid-*β* (A*β*) peptides in the brain. Amyloid precursor protein is cleaved by *γ*-secretases to generate A*β*, which can accumulate into a plaque, causing neurological deficits. In mouse studies, TMED10 expression was widespread throughout the grey matter of the brain, but it was predominantly localized to the presynaptic membranes of neuronal junctions. As amyloid precursor processing enzymes like *γ*-secretase were also localized to presynaptic membranes, the presence of TMED10 at presynaptic junctions suggests a role in A*β* secretion (Laßek *et al.*, 2013; Liu *et al.*, 2015). In addition, TMED10 also suppresses the transport of amyloid precursor proteins through the secretory pathway, resulting in less accumulation of fully processed amyloid precursor protein. TMED9 also reduced A*β* accumulation in the brain (Hasegawa *et al.*, 2010). Therefore, TMED9 and TMED10 might play an important role in preventing Alzheimer's disease pathogenesis in humans (Chen *et al.*, 2006; Hasegawa *et al.*, 2010; Liu *et al.*, 2015).

### 3.5. Liver disease

Work from our laboratory showed that *Tmed2* may play a role in the progression of non-alcoholic fatty liver disease (NAFLD) (Hou *et al.*, 2017), which is the major cause of chronic liver disease worldwide (Abd El-Kader & El-Den Ashmawy, 2015). Low TMED2 levels were associated with a corresponding decrease in TMED10 levels. In this novel NAFLD model, TMED2 was not required for activation of the tunicamycin-associated unfolded protein response. However, livers isolated from heterozygous mice had dilated ER membranes and increased levels of eIF2*α*, suggesting ER stress and activation of the PERK unfolded protein response (Hou *et al.*, 2017). Histological studies of mouse livers showed that 28% of heterozygous mice displayed clinical features associated with NAFLD by the age of 6 months.

## 4. Future directions

Our understanding of TMED proteins in development and disease is in its infancy, and in fact little is known regarding the primate-specific TMED11. These proteins are evolutionarily conserved, and their localization and levels are tightly regulated by the cellular machinery. As discussed, individual TMED proteins are important for morphogenesis, differentiation and homeostasis in a number of organisms (Carney & Taylor, 2003; Boltz *et al.*, 2007; Vetrivel *et al.*, 2008; Jerome-Majewska *et al.*, 2010; Graveley *et al.*, 2011; Zakariyah *et al.*, 2012; Darville & Sokolowski, 2018). However, although their roles in cargo transport are clearly important and numerous putative cargo proteins are regulated by TMED proteins (Tables 2 & 3), little is understood of the impact of most TMED proteins in development and disease. TMED proteins are found to interact with lipids and with high specificity for specific carbon chain length, begging the question of their contribution to the structure and integrity of the various organelles in which they are found. There are no unifying themes emerging on the requirement, localization or function of TMED proteins in the many organs in which they are expressed. In fact, current data challenge the longstanding notion that TMED proteins are redundant. Thus, it is imperative that individual TMED proteins be studied. In addition, TMED protein oligomerization – a longstanding assumption – is poorly understood and may also be tissue- and temporal-specific. The finding that loss of a TMED protein does not always result in loss of associating members makes a very strong case for studying how TMED proteins interact within different tissues and organisms. Finally, point mutations in the GOLD domain, deletions of large portions of the gene or premature stop codons before the carboxyl domain of TMED proteins all result in loss of function. Thus, it is enticing to speculate that variants found in human TMED genes may directly contribute to the diseases in which they are misregulated.
